# Circular economy priorities for photovoltaics in the energy transition

**DOI:** 10.1371/journal.pone.0274351

**Published:** 2022-09-09

**Authors:** Heather Mirletz, Silvana Ovaitt, Seetharaman Sridhar, Teresa M. Barnes

**Affiliations:** 1 Advanced Energy Systems Graduate Program, Colorado School of Mines, Golden, Colorado, United States of America; 2 National Renewable Energy Laboratory, Golden, Colorado, United States of America; 3 Arizona State University, Tempe, Arizona, United States of America; University of Calabria Faculty of Engineering: Universita della Calabria, ITALY

## Abstract

Among the many ambitious decarbonization goals globally, the US intends grid decarbonization by 2035, requiring 1 TW of installed photovoltaics (PV), up from ~110 GW in 2021. This unprecedented global scale-up will stress existing PV supply chains with increased material and energy demands. By 2050, 1.75 TW of PV in the US cumulatively demands 97 million metric tonnes of virgin material and creates 8 million metric tonnes of life cycle waste. This analysis leverages the PV in Circular Economy tool (PV ICE) to evaluate two circular economy approaches, lifetime extension and closed-loop recycling, on their ability to reduce virgin material demands and life cycle wastes while meeting capacity goals. Modules with 50-year lifetimes can reduce virgin material demand by 3% through reduced deployment. Modules with 15-year lifetimes require an additional 1.2 TW of replacement modules to maintain capacity, increasing virgin material demand and waste unless >90% of module mass is closed-loop recycled. Currently, no PV technology is more than 90% closed-loop recycled. Glass, the majority of mass in all PV technologies and an energy intensive component with a problematic supply chain, should be targeted for a circular redesign. Our work contributes data-backed insights prioritizing circular PV strategies for a sustainable energy transition.

## 1. Introduction

**Fig 1:**
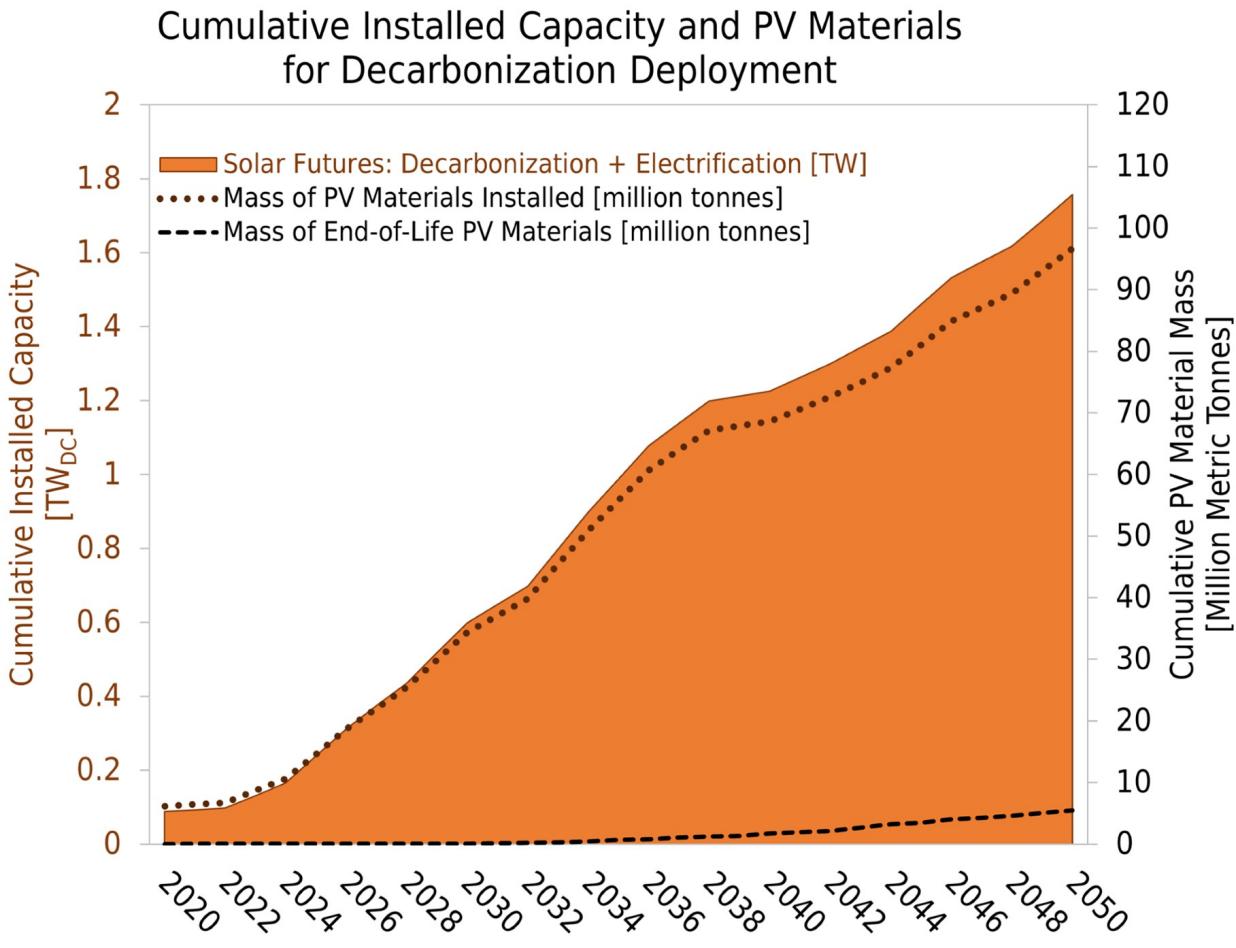
Installations, virgin material demands, and waste projection. Installation projections for photovoltaics (PV) in TW_DC_ as predicted in the Solar Futures Decarbonization + Electrification scenario [3] (left axis) for this US case study. The dotted line shows the cumulative mass of PV materials deployed (right axis). The black dashed line shows the cumulative PV end-of-life material projection through 2050 based on historical and expected technology trends as captured in the PV in circular economy tool (PV ICE) baseline (right axis). The Decarbonization + Electrification scenario relies on a least-cost optimization model to meet targets, resulting in a variable deployment rate [8].

Globally, many national decarbonization goals have been declared; for example, the United States (US) aspires to decarbonize the electricity grid by 2035 [1]. In 2020, the US added a record 19.2 GW of photovoltaics (PV) to the grid [2], bringing the cumulative capacity to 100 GW. Since 2020, multiple deployment models have been published all showing tremendous annual PV deployment growth to meet decarbonization targets [3–6]. US cumulative capacity needs to grow to ~1 TW in the next 15 years, which will require unprecedented increases in material use, manufacturing, deployment, grid integration, and eventually end-of-life (EoL) management. As a national decarbonization case study, we use data from the Solar Futures Study on the Decarbonization with Electrification scenario [3]. Fig 1 shows the cumulative nameplate installed PV capacity, the mass of installed materials, and the predicted mass of EoL materials necessary for US decarbonization deployment. Additionally, most fossil generation in the US is expected to be retired within the same time frame [7], which further adds to the urgency of the need for proactive planning to ensure a just and efficient energy transition.

A rapid increase in PV deployment rates requires us to address multiple challenges, including scaling up installation capability, maintaining installed capacity, sourcing materials from secure and sustainable supply chains, and responsibly managing manufacturing waste streams and decommissioned PV systems. PV systems with high yields and reliability are needed to reach and maintain necessary capacities. Increased reliability through proactive operations and maintenance (O&M) ensure maximum power production of PV systems [9]. Additionally, there are concerns about material shortages for achieving broad decarbonization and electrification globally [10–13]. For this, we will need to expand existing PV material supply chains, while improving their security and sustainability and reducing their negative environmental and social impacts [14–18]. Finally, deployed PV will eventually reach EoL and must be managed [19–21], although most of this will occur after 2050. Recovering PV materials could enable more secure and sustainable supply chains. We aim to address these challenges through a single dynamic model, which can assist with determining data-driven solutions and identifying potential tradeoffs.

The material requirements for a rapid energy transition can be mitigated by circular economy approaches, which can also enable a more resilient supply chain in the long term [3, 22]. Although definitions of circular economy differ, its practical implementation strives for materials recirculation throughout the economy to minimize virgin material extraction and landfilled waste. This can be achieved by prioritizing actions such as reduce, reuse, repair, remanufacture, and recycle [23, 24].

Several technology modifications could be made to PV to improve circularity, as covered by several studies [20, 25–28]. These could include increasing system lifetime and reliability [28–31], improving closed-loop module recycling [32, 33], or module remanufacturing [34–36]. Closed-loop recycling recovers PV materials for new PV modules, whereas downcycling recovers materials for other uses with less stringent quality specifications. However, there is a lack of analyses that quantify and compare the environmental impacts of different circular strategies [37]. Our study takes the first step, mass flow quantification, comparing circular strategies in a broad “what-if” set of scenarios.

This analysis leverages the PV in Circular Economy tool (PV ICE) to explore two potential circular technology designs or life cycle management options for PV: lifetime extension and closed-loop recycling. Both strategies will be evaluated on their ability to deploy and maintain decarbonization capacities as well as quantities of extraction and waste. PV ICE is a quantitative dynamic framework for evaluating PV module and system materials, manufacturing, design, and life cycle management options [38, 39]. PV ICE uses dynamic, user-defined module properties, component material composition, and deployment forecasts to calculate the effective capacity (defined below), virgin material demands, and life cycle wastes accounting for circular material flows.

This analysis spans the timeframe of 2010 through 2050 since most current deployment forecasts stop at 2050, which dictates the time window chosen here. Our research captures historically deployed modules and quantifies the challenge of decarbonization deployment rates in the US as a national case study; PV ICE can use any deployment schedule at any scale. We focus on PV modules, which constitute much of a PV system’s cost, mass, and near-critical materials. Given its market dominance, we only consider crystalline silicon (c-Si) technology. First, we explore the effect of PV module lifetime on effective capacity by varying module lifetime between 15 and 50 years, compared to a 35-year baseline. Then, we examine the impacts of two circular PV designs or life cycle management options—module lifetime and closed-loop recycling rate—on virgin material demands and life cycle wastes. Next, we observe to what extent EoL materials could offset the material needs of rapid deployment. Finally, we outline the next set of analyses necessary to holistically evaluate a circular economy for PV.

## 2. Effect of lifetime on PV capacity

Decarbonization requires aggressive PV deployment, and faster deployment rates will be the most material intensive. To represent the most challenging US case study, we selected the Solar Futures Decarbonization With Electrification (Decarb+E) [3] scenario to analyze in the PV ICE tool. The Decarb+E scenario assumes significant PV technology cost reductions, demand-side flexibility, and electrification of other sectors while optimizing for the lowest cost over all sectors [6]. The ReEDS least-cost optimization model results in a variable deployment curve for PV [8]. The Decarb+E scenario deploys a cumulative nameplate capacity of 1.75 TW_DC_ of PV by 2050, which is similar to other decarbonization studies, including Williams et al. 2021 (2.392 TW) [4] and Clack et al. 2020 (1.045 TW) [40]. However, Decarb+E achieves 95% grid decarbonization in 2035 instead of 2050, making it the most rapid US PV deployment projection (1 TW) for the next 15 years. We examine the impact of this rapid US PV deployment on capacity and module materials. In future works, we plan to consider other system components as well.

PV modules are typically the longest-lived component in a PV system and the most significant capital investment; hence, we use modules as a proxy for system lifetime. Our analysis compares module lifetime modifications against the PV ICE baseline, which is a dynamic historical average of c-Si module technology. Most deployment scenarios assume a constant lifetime, but c-Si PV module and system lifetimes have steadily increased, from around 15 years in the 1990s to between 32 and 35 years currently [41]. PV ICE baselines capture historical and expected technology changes and improvements, including lifetime, reliability, module efficiency, and material efficiency and composition [38]. Lifetime and reliability parameters are varied, while module and material improvements are identical across all scenarios; more details are available in the Methods section and in Ovaitt and Mirletz 2022 [38]. We use PV ICE baselines as our best conservative guess of future c-Si module properties and as a comparison point for our what-if analysis results.

We varied module lifetimes from 15 to 50 years, assuming identical annual deployment rates (a cumulative nameplate installed capacity of 1.75 TW), and examined the impact on effective PV capacity. Fifteen years has been proposed as the shortest PV module lifetime that is economically viable [42]. A 15-year module could also represent a module with planned obsolescence, poor quality [43], or site repowering, a practice where modules not yet at their technological EoL reach an economic EoL and are replaced with newer, more efficient modules [44]. Fifty-year PV modules are a target of the U.S. Department of Energy [45, 46].

**Fig 2:**
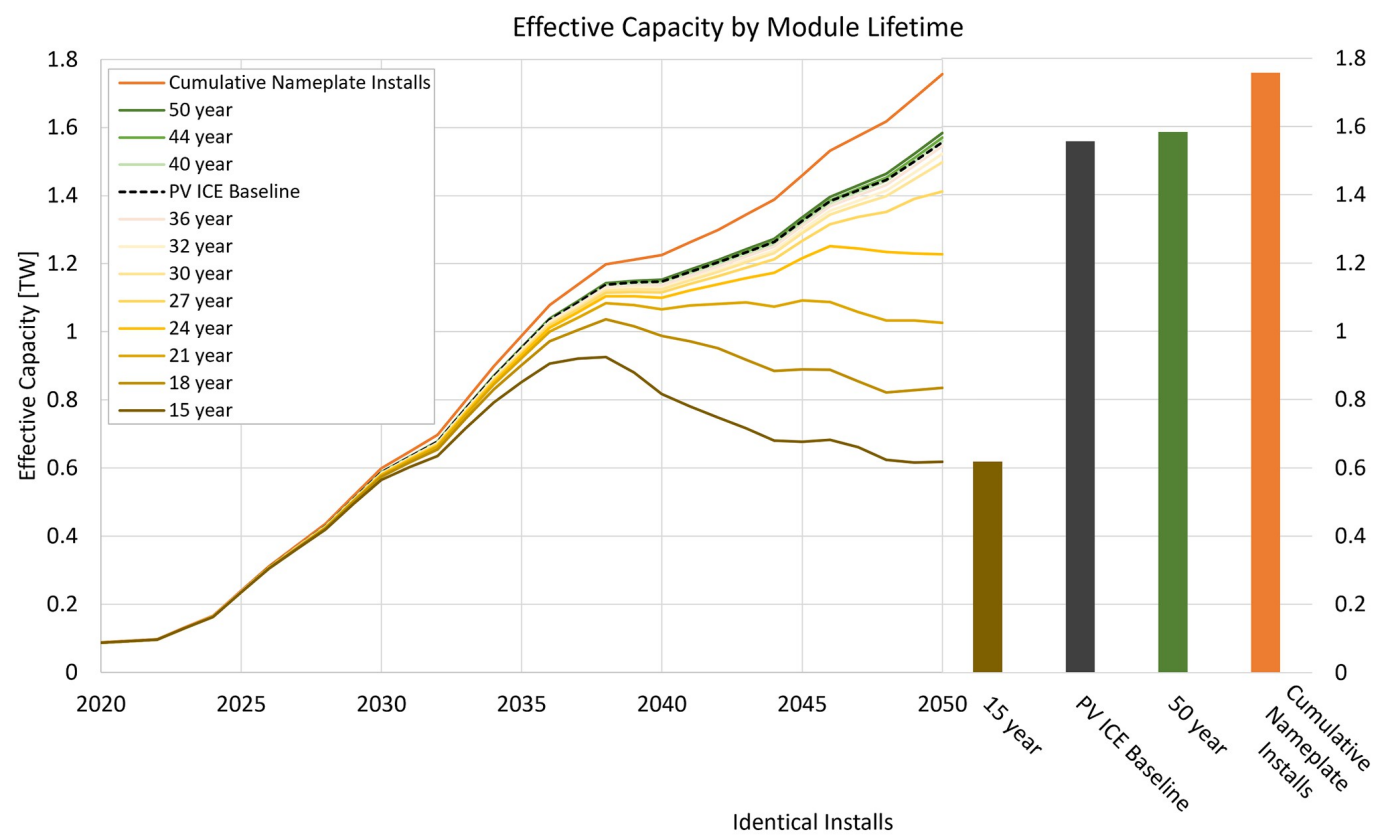
Effect of module lifetime and degradation on effective capacity. Effect of module lifetime on effective capacity, annually from 2020 through 2050, assuming identical deployment rates. The bar chart at the right shows the 2050 effective capacity of a 15-year module, the PV ICE baseline (a ~35-year module), and a 50-year module. The 1.75-TW cumulative nameplate installed capacity is provided as a comparison.

In this work, we define effective capacity as all cumulative nameplate installations (in this deployment case, 1.75 TW) minus failures, EoL retirements, and expected power degradation; this quantity is inherently cumulative and represents available generation capacity which will always be smaller than the nameplate installed capacity. Effective capacity calculations include continuing improvements in module efficiency and account for power degradation [38]. Fig 2 shows the effective capacity over time resulting from the Decarb+E deployment scenario [3] as a function of module lifetime without replacements. Each module lifetime scenario installs a total of 1.75 TW nameplate capacity (orange line and bar) between 2022–2050, identical to cumulative installs (orange) in Fig 1. The inflection observed after 2035 is a consequence of achieving 95% decarbonization within 15 years, and a slowing of deployment through 2050. The bar chart in Fig 2 compares the effective capacity in 2050 for a 15-year module, the PV ICE baseline (a ~35-year module), and a 50-year module, with the cumulative nameplate installed capacity as a reference comparison.

Effective capacity is strongly dependent on module lifetime. Compared to the PV ICE baseline (black dashed line), increasing module lifetimes to 50 years increases capacity in 2050 by 2%. Longer module lifetimes mean fewer modules will have to be replaced before 2050. Conversely, the decommissioning rate of short-lived modules outpaces the installation rate after 2035, resulting in massively decreased effective capacity. The effective capacity in 2050 can be reduced by more than 60%—1 TW—if a 15-year module is deployed without replacements. This is not conducive to rapid decarbonization since short-lived modules without replacements fail to maintain the necessary capacity. Therefore, if module lifetimes were to decrease, more replacements would need to be deployed. Note that this graph displays effective capacity alongside the 1.75-TW cumulative nameplate installed capacity; if an effective capacity of 1.75 TW is necessary, all scenarios may require some replacements.

**Fig 3:**
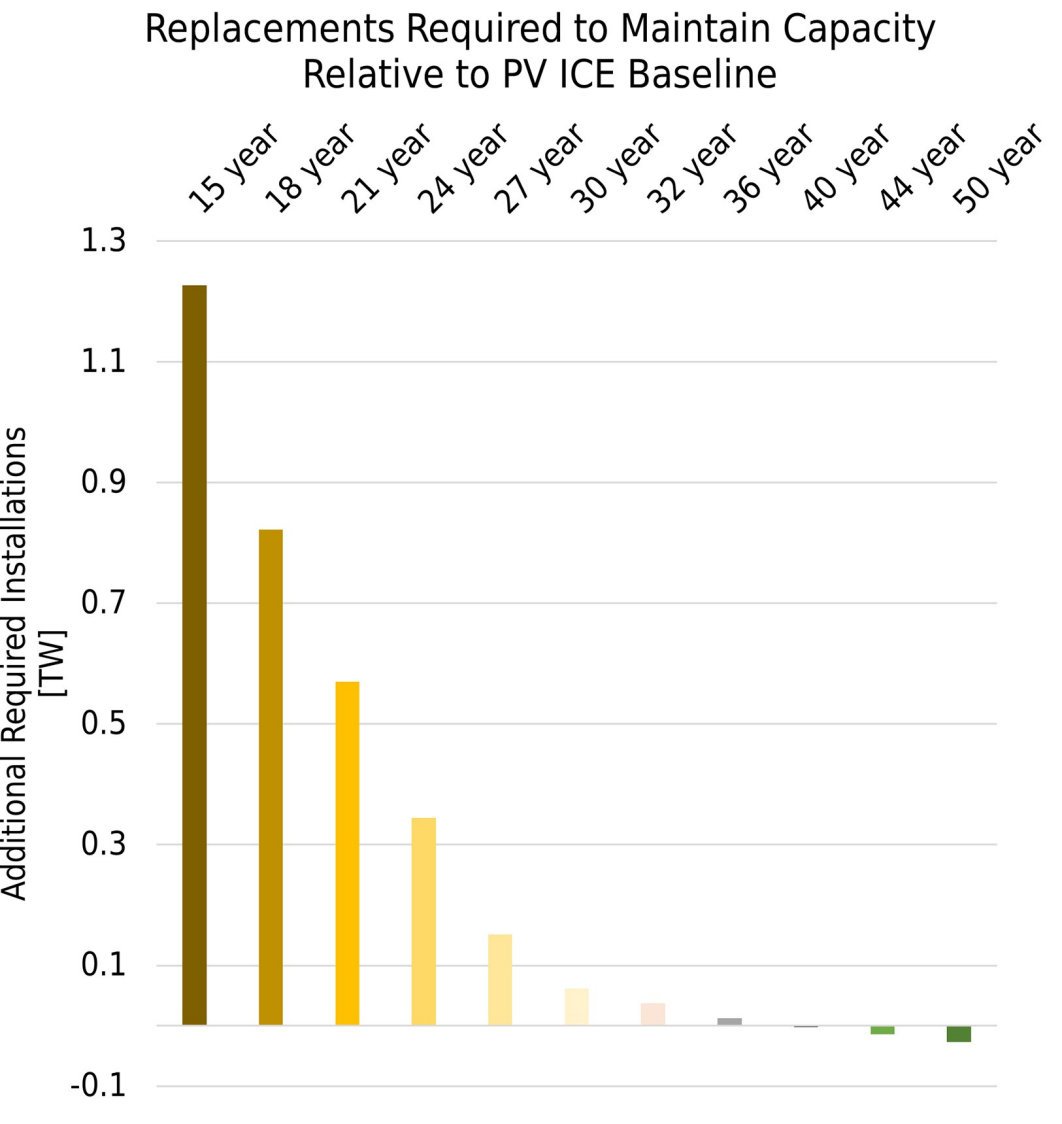
Cumulative required replacements. Cumulative additional nameplate installations required to replace failed modules and maintain effective capacity by module lifetime. The increase or decrease in required installations is relative to the effective capacity for the PV ICE baseline.

Given this shortfall in capacity, we compensated annual deployment rates with replacement modules to match the effective capacity of the PV ICE baseline (~35-year modules). Fig 3 shows the additional cumulative nameplate deployments required for different module lifetimes to maintain the PV ICE baseline’s effective capacity. A 50-year module could minimally reduce deployment requirements, whereas a 15-year module would increase deployment requirements by 1.2 TW. This increased deployment entails more virgin materials, life cycle waste, manufacturing infrastructure, labor, transportation, and EoL management for those replacement modules. Circular PV designs or life cycle management options could address some of these challenges.

## 3. Material impacts of PV lifetime and recycling

Although near the bottom of rank-ordered circular actions [47], recycling is touted as a popular antidote to raw material extraction [20, 48]. PV recycling is currently nascent but receiving much attention. The European Union’s Waste from Electrical and Electronic Equipment (WEEE) rules mandate that 80% of a PV module by mass must be recycled [49, 50]. In the US, most EoL c-Si PV modules end up in landfills, only one existing c-Si PV recycler recovers high-purity and bulk materials with a capacity of ~45 tonnes per day, and the quantity of EoL modules is poorly tracked [21, 51–53]. CdTe modules are already recycled with closed-loop recapture of the semiconductor [54]. Recycling is an important recovery mechanism and may be the only recourse for storm-damaged modules or obsolete low-power technologies.

As part of moving toward a circular economy, recycling processes should be improved as a tool to retain the value of materials we have mined, refined, and made useable for products. PV recycling is currently challenging due to costs, and no PV technology has demonstrated closed-loop recycling for all component materials. PV glass, which makes up the majority of any PV module’s mass, is a high-quality material with a problematic supply chain [15] and a high sensitivity to impurities during manufacturing [55–57]. However, glass manufacturing gleans energy and material savings during the melting process from the use of high-purity scrap (cullet) [58–60]. Unfortunately, glass from recycled PV modules (including CdTe) is downcycled to container glass or fiberglass due to potential contaminants. PV-quality glass represents a major opportunity to close the PV material loop.

Emerging technologies could design for circularity before commercialization [35, 42, 61]. For example, a 15-year module could be fully closed-loop recyclable, meaning that the same materials would be cycled repeatedly into new and improved modules every 15 years. But would this cycling of materials be sufficient to supply decarbonization deployment rates, including the extra 1.2 TW of replacements, while minimizing material extraction? Our objective is to evaluate the tradeoffs between designing for long or short lifetimes with fully closed-loop recycling. We did this by analyzing the boundary space of module lifetime and recycling rate, exploring increments between these edge case “what-if” scenarios.

Using PV ICE, we analyzed the effects of module lifetime and closed-loop recycling rate on PV virgin material demands and life cycle wastes for deployments, including replacements, as shown in Fig 3. We examined 336 combinations of module lifetimes from 15 to 50 years and closed-loop recycling rates from 0% to 100% in 5% increments and compared the results to PV ICE baseline predictions (~35-year lifetime and <15% downcycled). Life cycle wastes include manufacturing scrap as well as EoL wastes. Closed-loop recycling offsets and reduces virgin material demand and diverts waste from the landfill. All scenarios assume identical module efficiency improvements and bill of materials (BOM) to showcase and isolate the effect of lifetime and recycling. All scenarios also have identical pre-2020 US module deployment and US recycling rates to maintain as much historical accuracy as possible. The PV ICE Baseline BOM is available on Github [39], and further details can be found in the Methods section and in [38].

**Fig 4:**
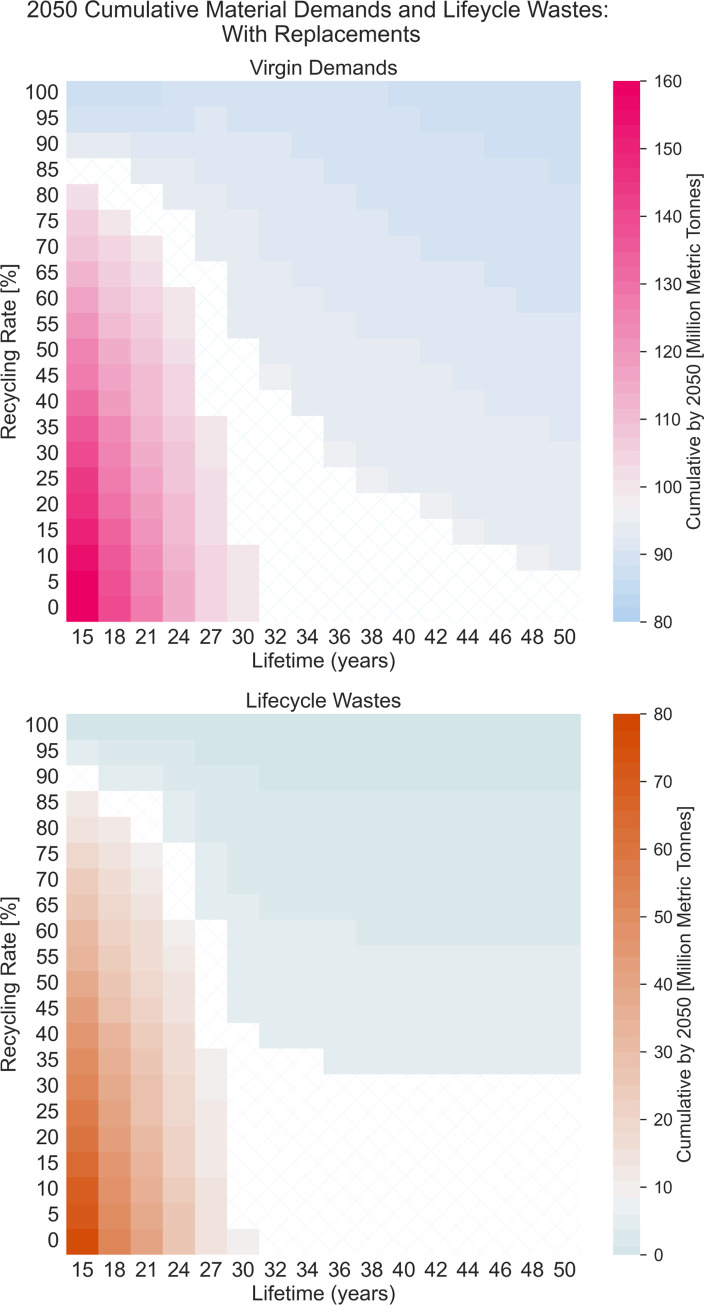
Lifetime vs recycling rate heat maps of virgin material demands and lifecycle wastes. Heat maps of (a) virgin material demands and (b) life cycle wastes in million metric tonnes for all combinations of module lifetime (x-axis) and closed-loop recycling rate (y-axis). Masses are cumulative from 2010–2050. Life cycle wastes includes manufacturing scrap and EoL wastes. This analysis accounts for replacement modules to maintain effective capacity. The white background with hashes indicates comparable mass to PV ICE baseline predictions, +/- 2 million metric tonnes. Color boundaries are in ~2–3 million metric tonne increments to delineate subtle differences.

Fig 4 shows the cumulative virgin material demands and life cycle wastes for all combinations of module lifetime and closed-loop recycling rate scenarios. The results—discussed further in the next two sections—show the cumulative virgin material needs and life cycle wastes from 2010 through 2050 in millions of metric tonnes. Heat map color bars are white with hashes for values comparable to PV ICE baseline predictions (+/- 2 million metric tonnes), blue/teal for less mass, and pink/red for more mass. These heat maps demonstrate that both parameters (closed-loop recycling rate and module lifetime) affect virgin material demands and life cycle wastes. Table 1 shows the cumulative (2010–2050) virgin material demands and life cycle wastes for the PV ICE baseline and selected results from the heat maps in Fig 4. The virgin material demands in Table 1 account for the offset provided by closed-loop recycled materials and include replacement modules to maintain effective capacity.

**Table 1:** Select cumulative virgin material demand and lifecycle wastes. Cumulative 2010 through 2050 virgin material demands and life cycle wastes of selected technology combinations. 100% recycling is closed-loop, which offsets virgin material demands.

Technology Scenario	Virgin Material Demands (million metric tonnes)	Life cycle Wastes (million metric tonnes)
PV ICE Baseline	97	8
50-Year Module, 0% Recycling	95	9
50-Year Module, 50% Recycling	91	4.5
50-Year Module, 100% Recycling	87	0.3
15-Year Module, 0% Recycling	162	76
15-Year Module, 50% Recycling	124	38
15-Year Module, 90% Recycling	94	8
15-Year Module, 95% Recycling	90	4
15-Year Module, 100% Recycling	87	0.3

### 3.1 Virgin material demands

Reducing virgin material extraction and refinement reduces a technology’s environmental footprint. The PV ICE baseline predicts 97 million metric tonnes of raw material demand cumulatively by 2050. Increasing module lifetime above 35 years slightly decreases the required virgin material due to the lower degradation and failure rates, requiring fewer installed modules to meet targeted capacities. Alternatively, deploying the same number of modules as the PV ICE baseline (using 97 million metric tonnes of PV materials) can yield a slight effective capacity boost, as seen in Fig 2.

A 15-year module, including 1.2 TW extra deployments to replace failed modules and maintain capacity, increases virgin material demands significantly, unless the closed-loop recycling rate is at least 90%. A 15-year module with 0% recycling would require nearly 70% more material than the PV ICE baseline’s virgin material extraction, up to 162 million metric tonnes. This emphasizes the importance of avoiding planned obsolescence. Decreasing the mass per module area (i.e., BOM) or raising the module nameplate efficiency will lower the closed-loop recycling rates required to reduce virgin material demands, as demonstrated in several sensitivity analyses [38, 62]. Overall, due to the scale of decarbonization, minimal reduction in virgin material demands is achievable before 2050 through modification of module lifetime and closed-loop recycling alone.

### 3.2 Life cycle wastes

Reducing wastes throughout the PV lifecycle lowers the environmental impact of the technology. The PV ICE baseline predicts 8 million metric tonnes of PV module wastes cumulatively by 2050, as shown in Table 1. This waste includes both manufacturing scrap and EoL waste. A longer-lived module is comparable to the PV ICE baseline, resulting in 9 million metric tonnes of life cycle wastes cumulatively by 2050. This waste can be decreased with closed-loop recycling rates above 30%. This is because almost all modules would still be active in the field in 2050, would have degraded less, and potentially fewer modules could be installed to maintain effective capacity (Fig 3). Therefore, life cycle wastes of long-lived modules before 2050 are attributable to manufacturing scrap, shorter-lived modules installed before 2020, and premature failures.

As shown in Fig 4, a 15-year module with >95% closed-loop recycled will produce less life cycle waste than the PV ICE baseline—as little as 0.3 million metric tonnes attributable to pre-2020 modules, which have low recyclability. However, if recycling rates of a short-lived module remain low, life cycle wastes could increase by two orders of magnitude, up to 76 million metric tonnes of waste (15-year, 0% recycled), which corresponds to the extra 1.2 TW needed to maintain capacity. For all PV modules that have shorter lifetimes than currently deployed technologies, this analysis demonstrates a threshold for closed-loop material recycling that can decrease life cycle wastes before 2050.

No current PV technologies have achieved greater than 95% closed-loop recycling for all component materials, although processes are proposed [63]. CdTe semiconductor material is above this threshold, but the glass (96% of CdTe module mass) and other component materials are downcycled out of the PV supply chain [64]. Alternate circular pathways, including remanufacturing, could enable higher levels of closed-loop material recirculation, but none are currently implemented for commercialized technologies.

### 3.3 Temporal variations in mass flows

Closed-loop material recycling requires the right quantities of materials to be available at the right time. One analysis (using no installed capacity targets) estimated that a successful closed-loop “cradle-to-cradle” PV materials system required module lifetimes of less than 10 years [65]. Required decarbonization deployment rates dictate the timing of material demand (i.e., decarbonization goals must be met), while module lifetime and reliability control when EoL materials become available to offset virgin material demand. Therefore, we examined the availability of EoL materials and manufacturing scrap compared to material demands through 2050. As previously mentioned, this study does not attempt to speculate past 2050. Still, we note that achieving a steady-state closed-loop material flow would depend on the post-2050 deployment rate.

**Fig 5:**
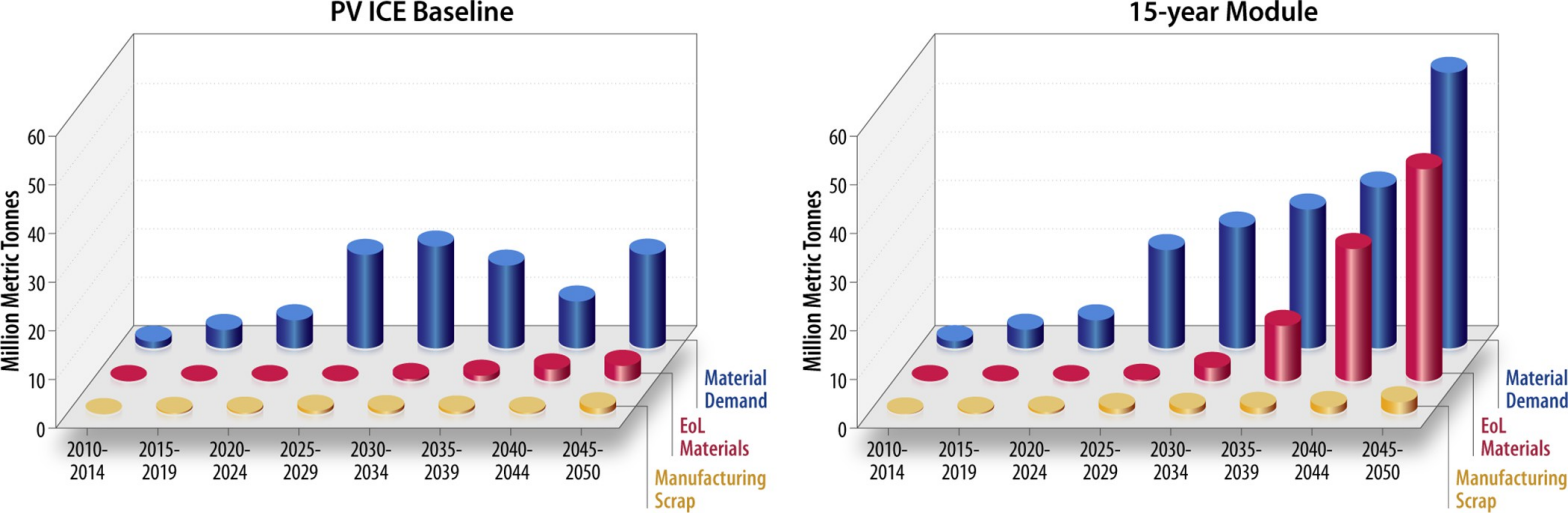
Time shift of material demand and EoL material availability. The 5-year summed material demand, EoL material, and manufacturing scrap for (a) the PV ICE baseline (~35-year module) and (b) a 15-year module. The material mass demands (blues) are the total material needed, which circular materials can offset. The EoL materials (red) and manufacturing scrap (yellow) are total material flows, assuming no circularity. All component materials are included.

Fig 5 shows the sum of annual material demands, manufacturing scrap, and EoL materials for every five years between 2010 and 2050, using historical deployments before 2020 and projected Decarb+E deployment from 2021–2050 (including the 1.2 TW of replacements). Fig 5 also illustrates the material demand (blue), EoL materials (red), and manufacturing scrap (yellow) for (a) the PV ICE baseline (~35-year lifetime) and (b) a 15-year module in 5-year increments in millions of metric tonnes.

To achieve 95% grid decarbonization by 2035, material demands of the PV ICE baseline peak at nearly 40 million metric tonnes between 2025–2029 and 2030–2034. EoL materials cannot offset more than 25% of material demand until after 2040 for >35-year modules. Cumulative EoL materials remain below 8 million metric tonnes until 2050—less than 10% of the cumulative demand. Although recovering valuable materials from PV modules is important, EoL recycling cannot offset the material demand of decarbonization for today’s modules because of this temporal offset.

The 5-year increment material demands of the 15-year module increase steadily to peak near 50 million metric tonnes between 2045 and 2050. This steady increase is due to the constant need to manufacture replacement modules. The 15-year module, the fastest material recirculation considered, does not see significant quantities of EoL material until after 2035. By 2050, the EoL material stream of a 15-year module will be well aligned in time with material demand, potentially enabling significant (but not 100%) offsetting of virgin material extraction. A rapid build out of US recycling infrastructure would be required to realize this virgin material offset; a processing capability of approximately 2 million metric tonnes per year by 2035–2040 and close to 7 million metric tonnes per year by 2045–2050 would be required to process both manufacturing scrap and EoL material of a 15-year module.

Manufacturing scrap is a material waste stream that is aligned in time with manufacturing demands. Although economic incentives are already aligned to encourage high manufacturing yields, manufacturing scrap forms an opportunity to retain the material value of extracted and refined materials. Manufacturing scrap recycling already exists for several PV component materials, including silicon kerf loss [66]. Some glass manufacturers have relationships with their customers to return low-contamination glass cullet, which, in addition to offsetting virgin materials, reduces the energy required to melt the sand and cullet [67, 68]. Manufacturing scrap will not offset all virgin material needs, but it provides a timely opportunity to retain material value and establish circular practices.

## 4. Methods

PV ICE [39] was leveraged with the Solar Futures [3] Decarbonization With Electrification (Decarb+E) US deployment scenario to analyze the mass flows and installed capacities for this national case study. Details of the PV ICE tool can be found in Ovaitt and Mirletz 2022 [38] and on the Github documentation [39]. A Jupyter journal of the analysis, “13—LifetimeVsRecycling Analysis–PLOS ONE,” is also available.

For this study, a PV ICE simulation calculated mass flows for 337 scenarios using the PV ICE baseline (see the PV ICE Baseline section) and all pairs of module lifetimes and recycling rates. The module lifetimes ranged from 15 to 30 years in 3-year increments and 30 to 50 years in 2-year increments. Recycling rates were incremented by 5%, from 0% to 100%. The annual mass flows of all scenarios were calculated using historical US deployment data for 2010 through 2020 and the annual deployment projections from Solar Futures [3] for 2021 through 2050. Installed capacity was allowed to vary. Next, the annual mass flows were recalculated, adjusting the annual PV installations to replace failed and EoL modules after 2020 (cumulative additional shown in Fig 3). Annual installations were adjusted relative to the PV ICE baseline effective capacity, revealing improvement or worsening relative to expected technology trends.

### 4.1 PV ICE baseline

**Fig 6:**
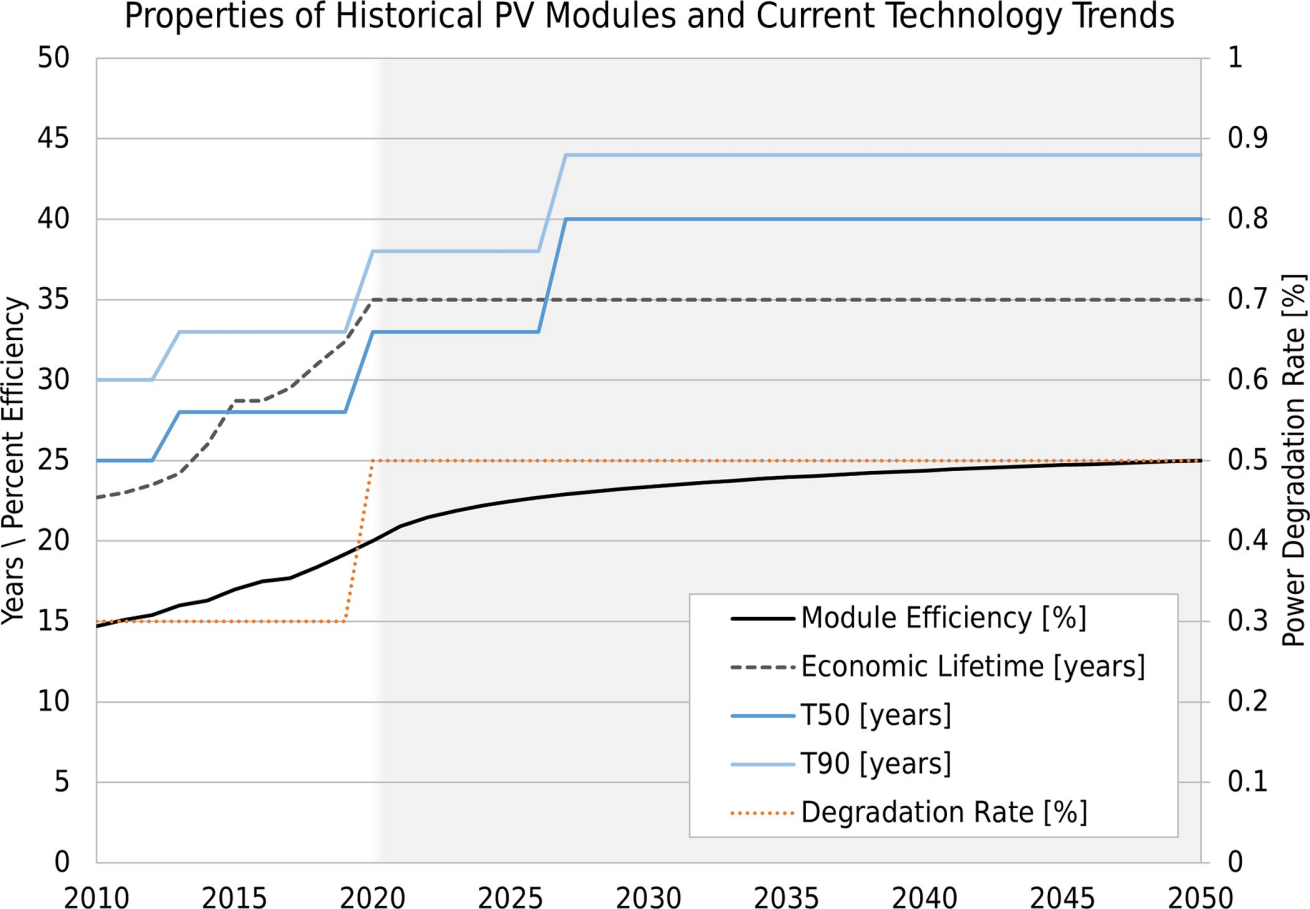
PV ICE Baseline properties. A selection of the dynamic module properties captured in the PV ICE baseline representing historical improvements and expected technology trends.

The PV ICE baseline is a set of historical averages of PV technology and expected future technology averages. Current PV modules have a ~32-year lifetime and are less than 15% open-loop recycled [38]. Our expected future technology improvements are conservative based on consensus reports [69]. The PV ICE baseline of module efficiency improvements was used for all scenarios. Fig 6 shows the average historical c-Si PV module lifetime properties and expected technology improvements. The average c-Si PV module efficiency will likely increase from 20% today to 25% by 2050 [70]. The economic project lifetime is expected to increase to 35 years around 2022 [41]. The degradation rate (right axis) is conservatively projected at 0.5%/year, although today’s modules currently average 0.3%/year [71]. The PV ICE degradation rate is lower than that used for a 36-year module (see Table 2). Finally, T50 and T90 are the time (in years) at which 50% and 90% of the modules have failed, respectively. These values control the shape of a Weibull probability function, a common tool in reliability science. The Weibull alpha and beta values corresponding to the PV ICE Baseline’s modern technology T50 and T90 values are 12.60 and 41.18, respectively. Other details of the dynamic average c-Si module baseline used in this study can be found in Ovaitt and Mirletz 2022 [38]. The technology modifications made in this analysis are relative to this PV ICE baseline. Our code and analysis are available on GitHub [39].

**Table 2:** Module lifetime parameters. Module lifetime parameters used to model modules of different lifetimes.

Project Lifetime	Degradation Rate	T50	T90	alpha	beta
[years]	[%/yr]	[years]	[years]		[years]
**15**	1.47	13.95	19.78	3.44	15.52
**18**	1.22	17.56	23.15	4.34	19.11
**21**	1.05	21.17	26.53	5.32	22.68
**24**	0.92	24.78	29.9	6.39	26.24
**27**	0.82	28.39	33.28	7.55	29.8
**30**	0.74	32	36.65	8.85	33.35
**32**	0.69	34.41	38.9	9.79	35.72
**34**	0.65	36.81	41.15	10.77	38.08
**36**	0.615	39.22	43.4	11.85	40.45
**38**	0.582	41.63	45.65	13.02	42.82
**40**	0.555	44.03	47.9	14.25	45.18
**42**	0.525	46.44	50.15	15.62	47.54
**44**	0.505	48.85	52.4	17.11	49.91
**46**	0.48	51.25	54.65	18.69	52.26
**48**	0.46	53.66	56.9	20.48	54.63
**50**	0.445	56.07	59.15	22.45	56.99

The same seven component materials and their properties based on average c-Si modules were used across all scenarios. Historical and future improvements in component material mass per module area are considered (e.g., reduction in silver per cell, thinner silicon cells). Our study is focused on the impacts of changing module lifetime and recycling and does not attempt to guess what technology changes might be entailed; therefore, no modifications are made to component materials or their properties. Our sensitivity analysis of the PV ICE tool shows that changes in mass per module area and module efficiency have a 1:1 effect on virgin material demands, life cycle wastes, and installed capacity [38, 62].

### 4.2 Varying module lifetime and closed-loop recycling rate

Four parameters control module lifetime and reliability in PV ICE: the economic project life, power degradation, and the T50 and T90 values of the Weibull probability function [38]. The economic project life was set to the range of module lifetimes. Module degradation rates were manually calculated using Eq (1) such that the module would produce 80% +/- 0.1% of nameplate power at the module EoL. The T50 and T90 values were calculated in Python using a linear regression such that only 10% of modules suffer failure by the project lifetime. Table 2 documents these parameters. For comparison, the Weibull parameters as made standard by [72] derived from [73], are alpha = 2.4928, beta = 30 for the early loss scenario and alpha = 5.3759, beta = 40 for the regular loss scenario.


$$\%\;of\;Nameplate\;Power=100*{\left(100-degradation\;rate\right)}^{year}$$ (1)

Recycling rates are controlled by both module and material properties in PV ICE. For this analysis, we assumed that all material flows were closed-loop, and recycled materials offset virgin material demand for new PV modules or were landfilled as waste. Furthermore, we assumed that all manufacturing scrap and EoL modules and materials were 100% collected, 100% dispositioned for recycling, and 100% recycled into high-quality closed-loop material, offsetting virgin material demand. The yield of the recycling process determined the closed-loop recycling rate, from 0% to 100%. Recycling rates are a combination of collection and process efficiency (e.g., 50% collection and 90% recycling process efficiency is a 45% recycling rate). We acknowledge that 100% closed-loop recycling is thermodynamically impossible and use it as a boundary space edge, a best-case scenario for a high-yield, high-value recycling process.

### 4.3 Data availability

The analysis presented here is publicly available in the Jupyter journal “13—LifetimeVsRecycling Analysis—PLOS ONE” on Github [39].

### 4.4 Limitations

We assumed identical module efficiency improvements and BOM (expressed as component material mass per module area). These assumptions effectively isolate the effects of changing PV module lifetimes and recycling rates but neglect any underlying technology changes that may be required. These two properties have been shown to have a 1:1 effect on the virgin material demands and life cycle wastes [38]. In a sensitivity analysis, we found that decreasing the mass per module area (BOM) and/or raising the module nameplate efficiency lowers the closed-loop recycling rate required for reducing virgin material demands to 80% and 60% respectively [62]; this analysis is documented in the Jupyter journal. Lifetime extension and closed-loop recycling could be achieved by changes to PV technology design, changes to PV life cycle management, or (likely) a combination of both. Emerging tandem silicon devices might have a material mass per module area similar to current silicon technologies, with a higher nameplate module efficiency. Given that there are no commercialized BOM or efficiencies to draw upon, we use these assumptions to highlight the effects of closed-loop recycling and lifetime and note how changes might affect the results. We do not attempt to predict how these changes will be made but focus our examination on the effects of these changes.

This study only considers 2010 through 2050. A 50-year module installed in 2050 would not be decommissioned until close to 2100; therefore, our cumulative by 2050 life cycle waste results exclude EoL wastes from these modules. However, few deployment projections extend to 2100, and this study does not attempt to predict post-2050 required capacities or deployment rates. Any extension of existing projections risks compounding assumptions. This study focuses on the mass flows between today and 2050, representing the most challenging timeframe in global decarbonization.

This study explores 100% closed-loop recycling; however, we acknowledge the thermodynamic impossibility of 100%. The intent is to use 100% as an idealized circular system or a best-case scenario proxy. Our purpose is to explore how a nearly ideal system might impact virgin material demands and life cycle wastes for energy transition and decarbonization. This study does not attempt to guess which underlying technology changes or processes will achieve high-yield, high-value recycling or module lifetime extension. The PV ICE framework can explore specific design modifications, gradual changes, or policy changes.

## 5. Conclusions

This boundary space “what-if” analysis explores the capacity and material effects of circular pathways for PV, module lifetimes, and closed-loop recycling, considering pre-2050 decarbonization deployment rates for a national-level case study of the US. These aggressive deployment rates create challenges in scaling up manufacturing, installation, maintaining capacity, responsibly sourcing materials, and managing EoL. From the results of our 336 lifetime-recycling scenarios, we find that on a mass basis there are multiple ways to reduce virgin material demands and life cycle wastes while maintaining installed capacity through 2050. A long-lived module, for example, can reduce virgin material demands and life cycle wastes through reduced deployment needs—the modules fail less often and degrade more slowly meaning fewer modules are required to meet a targeted capacity. These longer-lived modules also potentially provide a “grace period” to evaluate and implement responsible EoL management and build out necessary infrastructure; a 50-year module deployed today will not be decommissioned until 2070.

Increasing manufacturing scrap recovery is one promising pathway to reduce virgin material inputs with minimal collection costs; it is aligned temporally with demand and can potentially develop and refine methods for EoL recovery. Fig 4 shows that even modest closed-loop recycling rates (>30%) at current module lifetimes of ~35 years provide opportunities to reduce material demands and life cycle wastes; thus, we should strive to recover valuable PV materials. Even if closed-loop recycling for PV proves unachievable, recovery of these high-value materials (glass, silicon, silver) could benefit other sectors. Cross-industry material flow analysis is outside our scope but would be a valuable planning tool for implementing a circular economy.

Short-lived modules steepen the challenging deployment curve of energy transition, requiring up to 1.2 TW of replacement modules to maintain capacity through 2050. Due to this increased deployment, a short-lived PV module can only reduce virgin material demands and life cycle wastes if 95% closed-loop recycling is maintained. This requires 100% collection and a high-yield, high-value recycling processes, which presents a technology and management challenge because no PV technology has achieved this level of closed-loop recycling for all component materials. Currently, CdTe thin films can be closed-loop recycled; however, the flat glass, which represents the biggest opportunity by mass and a significant challenge to meet stringent quality requirements, is downcycled [64, 74]. One hundred percent (100%) EoL collection poses a challenge for distributed or unregulated PV, and currently, in the US, most EoL c-Si PV is landfilled [52]. Alternate material recirculation methods, such as material remanufacture, supported by circular business paradigms or policy mandates like the EU WEEE directive could help reach these challenging collection thresholds.

Our boundary space “what-if” analysis results from our 336 lifetime-recycling scenarios demonstrate that increasing PV module lifetimes and/or increasing closed-loop recycling rates can achieve reductions in virgin material demands and life cycle wastes while meeting the deployment rates required for decarbonization. Lifetime extension, for PV modules and systems, could be accomplished in several ways, such as continued module reliability and resiliency improvements. Research and design priorities for lifetime extension include refined accelerated testing protocols, manufacturing quality control, predictive modeling to assess the impacts of design changes, continuing efficiency and material improvements, forward and backward compatibility of modules and components, and repair and refurbishment of system components [43]. Offsetting virgin material demands can be accomplished in ways other than recycling, including high-yield, high-efficiency, reliable systems (thereby reducing replacement and total deployment needs); remanufacturing of components; and circular material sourcing. Other studies have cataloged detailed considerations of PV design for circular economy, current recycling status and challenges [25, 27, 75–77]. In addition to these technological circular economy designs, policy and business paradigms could support a circular economy for PV [78, 79]. A broader variety of circular options will be explored in future work.

**Fig 7:**
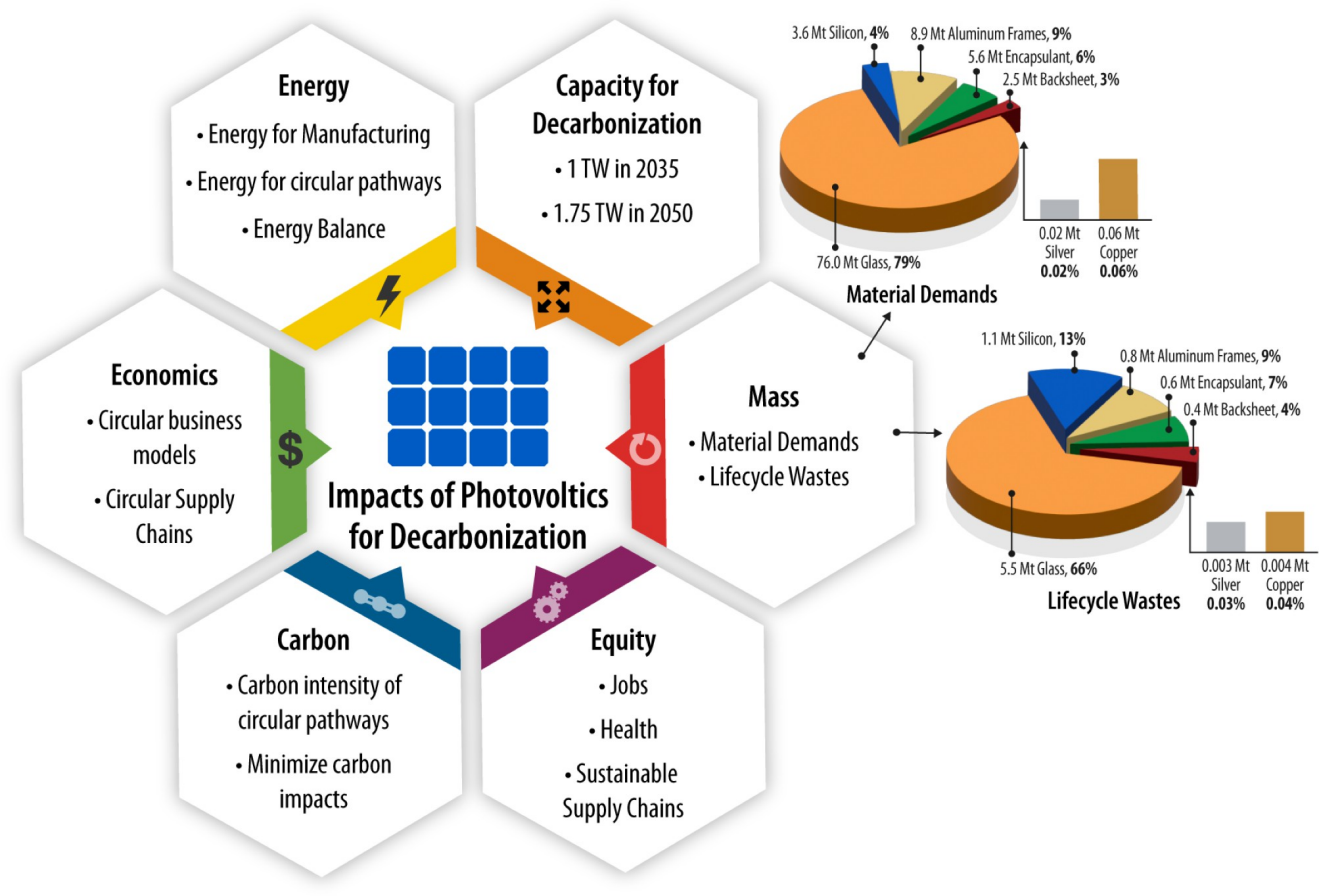
Holistic dimensions to evaluate circular PV for decarbonization. Dimensions and metrics for holistically evaluating circular PV design and life cycle management. The pie charts present the component material breakdowns of material demands and life cycle wastes for expected technology trends (PV ICE baseline) through 2050. Mt is million metric tonnes.

Glass is the most significant mass component in all PV technologies, and trends in c-Si modules have been toward more glass-glass packages [69]. Glass comprises 79% of the material demand and 67% of life cycle wastes, as seen in the pie charts in Fig 7. Therefore, glass forms a significant opportunity for rethinking and redesigning toward circularity. Options could include reducing glass thickness (while mitigating the reliability risk due to loss of mechanical strength), remanufacturing glass [36, 80–82], and recycling the cullet back into solar or flat glass quality instead of downcycling. Similarly, identifying non-extractive supply chains for PV component materials could increase supply while reducing environmental and social degradation [14, 15].

This first analysis is only on a mass basis, which does not provide a complete picture of sustainability [83, 84]. A more holistic set of dimensions for assessing sustainability is presented in Fig 7. As exemplified by the proposed EU Ecolabel Environmental Impact Index [85], energy generation and embodied energy must be considered for a thorough evaluation of energy generation technologies in addition to metrics of circularity, such as recyclability. For example, in our case study, the energy demands of PV module lifetime extension and closed-loop PV module recycling are likely to differ. Additionally, analysis of a 15-year module needs to account for the increased manufacturing energy for 15-year module replacements, as well as increased effluents, labor, transportation, and infrastructure. Finally, for decarbonization, renewable energy technologies must also reduce their carbon intensity. Therefore, our future work will conduct analyses of energy flows, environmental impacts (with a focus on carbon intensity), and socioeconomic impacts.
